# Adaptive Layering Algorithm for FDM-3D Printing Based on Optimal Volume Error

**DOI:** 10.3390/mi13060836

**Published:** 2022-05-26

**Authors:** Ning Lv, Xuefeng Ouyang, Yujing Qiao

**Affiliations:** 1 School of Mechanical Engineering, Yangzhou Polytechnic College, Yangzhou 225009, China; ning_lv@yzpc.edu.cn; 2 School of Automation, Harbin University of Science and Technology, Harbin 150080, China; ouyangxuefeng0451@126.com

**Keywords:** 3D printing, fused deposition modeling, step effect, optimal volume error, adaptive layering

## Abstract

The characteristics of fused deposition 3D printing lead to the inevitable step effect of surface contour in the process of forming and manufacturing, which affects molding accuracy. Traditional layering algorithms cannot take into account both printing time and molding accuracy. In this paper, an adaptive layering algorithm based on the optimal volume error is proposed. The angle between the normal vector and the layering direction is used for data optimization. The layer thickness is determined by calculating the volume error, and based on the principle of the optimal volume error, the unequal thickness adaptive layering of each printing layer of the model is realized. The experimental results show that the self-adaptive layering algorithm based on the optimal volume error has a better layering effect, greatly improves the forming efficiency and surface forming accuracy, and has a good adaptability to models with complex surfaces.

## 1. Introduction

In recent years, additive manufacturing technology has developed rapidly, and its application fields have become increasingly more extensive [1,2,3]. Fused Deposition Modeling (FDM) is one of the important processes of 3D printing [4,5]. Its process flow is as follows: The material moves along the section contour and filling track of the part after being heated and melted by the extrusion head. Then, the melted material is superimposed layer by layer with a certain layer thickness [6,7,8]. After the material is cooled and solidified, the supporting structure is removed to obtain the finished part. Layered slicing is an important step before the start of FDM-3D printing, and it converts 3D digital models into layered printing slices [9,10]. This specific method is used to intersect the plane perpendicular to the *z*-axis and the model grid in order to obtain the profile information of each layer of the model. The forming accuracy of 3D printing is closely related to layered planning, and the quality of the layered processing algorithm directly affects the forming quality and accuracy of the printed parts [11,12,13]. Since FDM is a layer-by-layer stacking process, there will be a step effect between layers along the *z*-axis direction. A smaller layer thickness can reduce the step effect and improve surface accuracy, but the molding time will also increase accordingly [14,15,16]. If considering the forming efficiency of parts, a thicker layer will lead to an obvious step effect and lower the precision of the finished parts [12,17].

The contradiction between the printing time and the printing accuracy can be effectively adjusted by layered slicing with unequal thicknesses [18]. There are two main methods commonly used: In the first method, a surface layer is adopted in areas with surface features of the model surface contour, and a plane layer is adopted in common parts [19]. The purpose of the other method of unequal thickness layering is to use the tangent angle of points on the vertical slice outline to determine the slice thickness after extracting the vertical outline of the model [20]. The hierarchical method based on model features has also been studied in recent years, but there are limitations in the definition of feature points, and it is difficult to identify complex surfaces. The analysis of the literatures show that the current research on the unequal thickness adaptive layering algorithm mainly focuses on how to reduce the step error of the model while reducing the number of layers as much as possible, but there is less research on the loss of model features affecting printing accuracy [21,22]. To solve the above problems, an FDM adaptive layering algorithm based on the optimal volume error is proposed in this paper. Based on the principle of the minimum stratification of the volume error, our algorithm optimizes triangular patches, selects the triangular patches required for adaptive layering, and then calculates the minimum volume error to determine the optimal layer thickness. Our algorithm not only considers a reduction in the step effect but also achieves accurate recognition and the retention of model features. Therefore, the proposed layered 3D printing algorithm greatly improves printing efficiency on the premise of ensuring printing accuracy, and it provides a certain theoretical and technical value for the further development of 3D printing.

In this paper, the hemispherical model and the turbine blade model are used in the experimental design to verify the adaptability of the algorithm and the application effect in practical engineering, respectively. The corresponding conclusions are given.

The rest of this paper is organized as follows: In Section 2, the FDM layering principle based on the optimal volume error is described, and the layer height of each layer is calculated. In Section 3, the FDM adaptive layering algorithm based on the optimal volume error is proposed to determine the optimal value of the current layering. Section 4 describes the implementation process of the proposed hierarchical algorithm. In Section 5, the layering algorithm proposed in this paper is used for experimental comparison and verification, and the corresponding conclusions are drawn.

## 2. The Principle of FDM Layering Based on Optimal Volume Error

**Figure 1:**
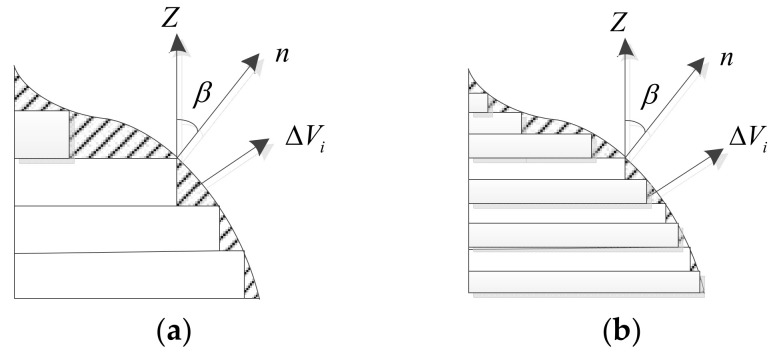
Step effect of FDM-3D printing. (**a**) The large volume error $\Delta V_{i}$. (**b**) The small volume error $\Delta V_{i}$.

When an FDM-3D model is used to print parts, the step effect will occur when two adjacent layers of orthographic projection do not coincide as shown in Figure 1, where *n* is the normal vector of the triangular facet, *β* is the angle between the normal vector of the model slice triangular patch and the *z*-axis, and $\Delta V_{i}$ is the volume error. The shaded part in Figure 1 is the error caused by the step effect, which will increase the surface roughness of the workpiece and have a greater impact on the overall accuracy. It can be seen that Δ*V* is smaller when *β* is larger. However, *β* is determined by the contour of the model and cannot be changed for a certain triangular patch. By comparing Figure 1a and Figure 1b, we can see that Δ*V* can be reduced by reasonably changing the layer thickness, which can improve printing accuracy. However, a reduction in the layer thickness will lead to a corresponding increase in the printing time.

**Figure 2:**
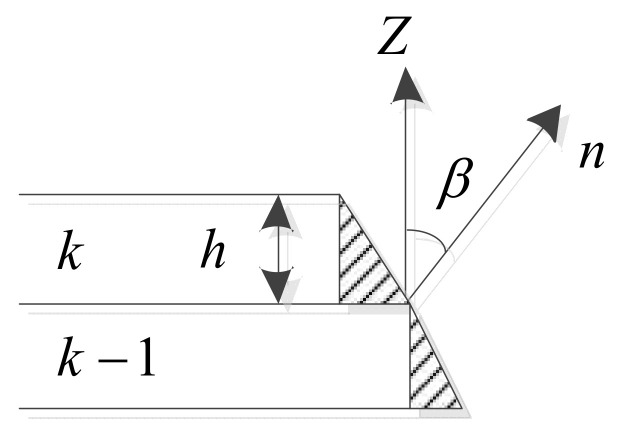
Schematic diagram of volume error.

When the printing layer height *h* is changed, the corresponding volume error will also change, so the approximate minimum volume error of each layer can be selected as the layering optimization goal. As shown in Figure 2, the projected area can be approximately regarded as a triangle in the calculation of the volume error, and then the cross-sectional area *S_i_* of the *i*-th triangular sheet step effect at the *k*-th layer can be expressed as Equation (1):

(1) $$Si\approx \;\frac{h^{2}}{2\;\tan\;\beta_{i}}$$

**Figure 3:**
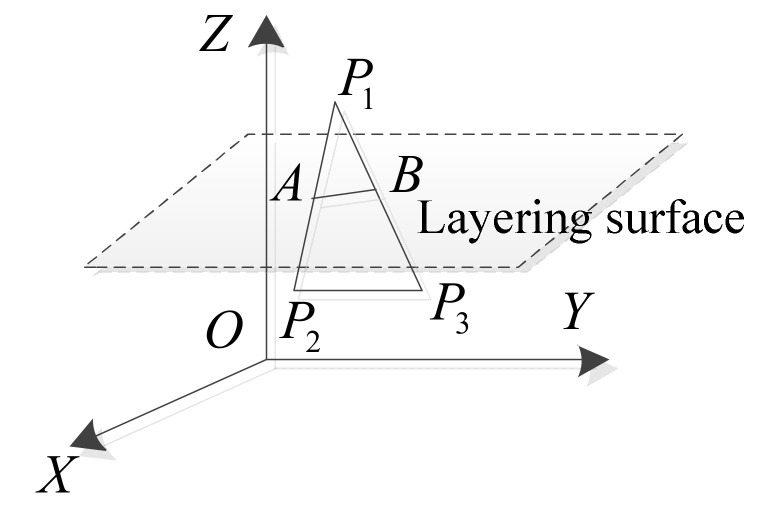
Schematic diagram of sliced triangular faces.

The error of each layered step is defined as Δ*V*, which can be regarded as a truncated cone surrounded by a plurality of triangular prisms, and the height of the triangular prisms forms the intersection line between the triangular plane and the layered plane, as shown in Figure 3.

The three vertices of the triangular patch in Figure 3 are $p_{1}\left(x_{1},y_{1},z_{1}\right)$, $p_{2}\left(x_{2},y_{2},z_{2}\right)$, and $p_{3}\left(x_{3},y_{3},z_{3}\right)$. Points A and B are the points where the triangular patch intersects with the layered surface. The current height of the two points A and B is *z*. The coordinates are A$\left(x_{a},y_{a},z\right)$ and B$\left(x_{b},y_{b},z\right)$. According to the geometric relationship, the coordinates of points A and B can be expressed by Equations (2) and (3):

(2) $$x_{a}=\frac{\left(z-z_{1}\right)\left(x_{1}-x_{2}\right)}{z_{1}-z_{2}}+x_{1};\;y_{a}=\frac{\left(z-z_{1}\right)\left(y_{1}-y_{2}\right)}{z_{1}-z_{2}}+y_{1}$$

(3) $$x_{b}=\frac{\left(z-z_{1}\right)\left(x_{1}-x_{3}\right)}{z_{1}-z_{3}}+x_{1};\;y_{b}=\frac{\left(z-z_{1}\right)\left(y_{1}-y_{3}\right)}{z_{1}-z_{3}}+y_{1}\;$$

The length of AB can be expressed by Equation (4):

(4) $$l_{AB}=\sqrt{{\left(\frac{\left(z-z_{1}\right)\left(x_{1}-x_{2}\right)}{z_{1}-z_{2}}-\frac{\left(z-z_{1}\right)\left(x_{1}-x_{3}\right)}{z_{1}-z_{3}}\right)}^{2}+{\left(\frac{\left(z-z_{1}\right)\left(y_{1}-y_{2}\right)}{z_{1}-z_{2}}-\frac{\left(z-z_{1}\right)\left(y_{1}-y_{3}\right)}{z_{1}-z_{3}}\right)}^{2}}$$

According to Equation (1) and Equation (4), the volume error of the *k*-th layer can be obtained as follows:

(5) $$\Delta V_{k}\;\approx \;\sum_{i=1}^{n}l_{i}S_{i}\;\approx \;\sum_{i=1}^{n}l_{i}\frac{h^{2}}{2\;\tan\;\beta_{i}}$$

where *n* is the number of triangular patches in the current layer.

The overall volume error of the STL model caused by the layered step effect is as follows:

(6) $$\Delta V\approx \sum_{m=1}^{m}\Delta V_{m}\approx \sum_{m=1}^{m}\sum_{i=1}^{n}l_{i}\frac{h_{m}{}^{2}}{2\tan\beta_{i}}$$

where *m* is the total number of STL layers. Taking the optimal volume error minΔV as the target, the height of each layer can be calculated.

## 3. Adaptive Layering Algorithm of FDM Based on Optimal Volume Error

**Figure 4:**
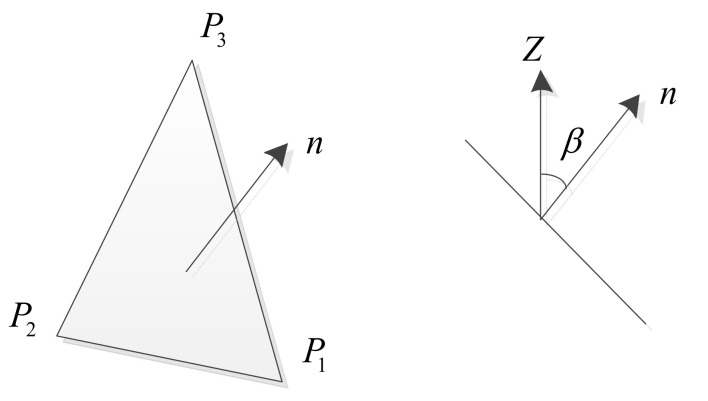
Schematic diagram of normal vector of triangle face.

FDM-3D printing adopts the STL file format for slicing, which is a data set of multiple triangular slices obtained by discretizing the surface of the CAD model [23]. The layering process based on the optimal volume error is also calculated based on the triangular patch set of the model. Firstly, the normal vector of the triangular patch is calculated, and the three vertices $p_{i}\left(x,y,z\right)$ of any triangular patch in the current layer are extracted, $\left(i=1,2,3\right)$, as shown in Figure 4. Three vertices are used to calculate the normal vector *n* of the triangular patch, as shown in Equation (7):

(7) $$\vec{n}=\vec{\left(p_{2}-p_{1}\right)}*\vec{\left(p_{3}-p_{1}\right)}$$

Equation (8) is used to calculate the angle *β* between the normal vector and the *z*-axis direction:

(8) $$\beta {=\cos}^{-1}\frac{\vec{n}\vec{z}}{\left|\vec{n}\right|\left|\vec{z}\right|}$$

When the angle β′>90° between the normal vector $\vec{n}$ and the *z*-axis, then β =180°−β′. If *β*′ = 0° and it is a horizontal triangle, then delete this triangle. The value range of *β* is *β* ∈ (0, π/2].

The layer thickness is preset before the layer calculation. It can be seen from the analysis of the triangular patch and step effect error that, when *β* is smaller, the step effect error is larger; that is, when the angle of *β* is small, the curvature of the model is larger, and the layer thickness should be smaller. When the angle of *β* is large, the curvature of the model is smaller, and the layer thickness can be appropriately increased. According to the above relationship, the function Equation (9) can be obtained:

(9) y=1−cosβ

where *β* is the angle between the normal vector and the axis, and *y* is the calculated interval ratio.

**Figure 5:**
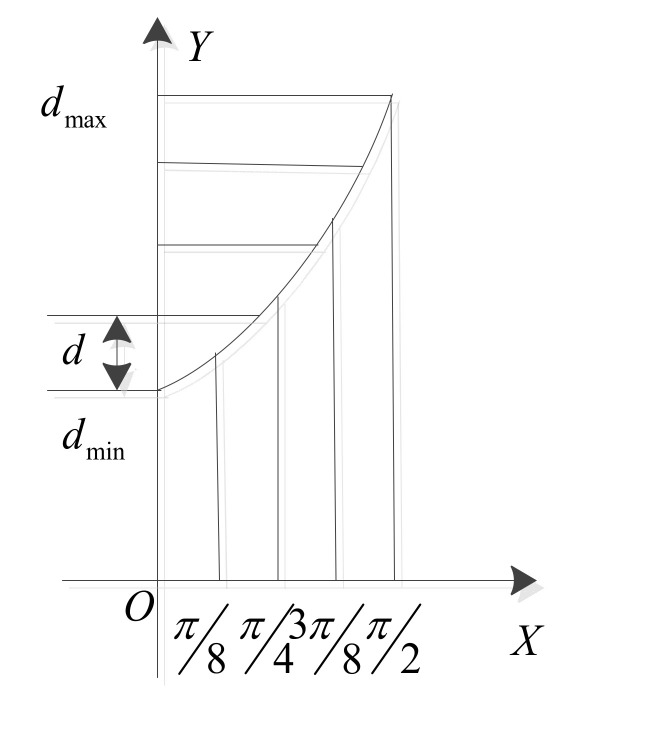
Schematic diagram of layer thickness.

The corresponding relationship between the function curve and the layer thickness is shown in Figure 5. Firstly, the layer thickness is set on the closed interval $\left[d_{\min},d_{\max}\right]$, and then, according to the angle of *β* and Equation (9), the relational formula of the layer thickness can be expressed as follows:

(10) $$h=(d_{\max}-d_{\min})(1-\cos\beta )+d_{\min}$$

According to the preset layering, the thickness corresponding to each triangular patch can be calculated, and the optimal volume error Equation (11) for the calculation of the current layer can be obtained through the deformation of Equation (5):

(11) $$\Delta V_{k}\approx \sum_{i=1}^{n}l_{i}S_{i}\approx \sum_{i=1}^{n}l_{i}\frac{h_{i}{}^{2}}{2\tan\beta_{i}}$$

## 4. Adaptive Layering Algorithm Flow of FDM Based on Optimal Volume Error

During FDM-3D printing, the initial layer generally has an edge support structure ensuring a reliable attachment between the model and the construction platform so as to avoid warping deformation [24]; therefore, it is not suitable for adaptive unequal thickness layering. The starting layer is sliced with a fixed thickness, which forms the supporting edge structure. From the second layer, the adaptive layered slicing based on the optimal volume error is performed, and the process is as follows:

Step 1: On the basis that the *z*-axis height completes the current layering, traverse all the triangular patches with the maximum layering thickness, filter out all the triangular patches that intersect with the current maximum layer thickness, and store all the screened triangles in array A.

Step 2: According to the size of the included angle *β*, bubble sort the data in array A from small to large to obtain array B. Using reservoir sampling, select the first *k* items in array B to form array C (*k* is 70% of the total data in array B), add the *k* + 1 item to array C, delete the first item, update the array C if the interval decreases, add *k* + 2 item if the interval does not decrease, delete the first two items, and judge whether the interval decreases, etc. When the interval is updated, add the first item after array C. Repeat the above process until the traversal of array B is complete and a new array C is obtained.

Step 3: Determine the different layer thicknesses at different slopes according to the angle *β* between the triangular facet normal vector and the *z*-axis in array C. Substitute the angle *β* and the corresponding thickness *h* into Equation (11) to determine the optimal volume error value, and then substitute the optimal volume error value and the included angle *β* into Equation (5) to inversely find the optimal thickness *h* of the current layer.

Step 4: Traverse array A again according to the current layer thickness *h*, delete the triangular patches that are not under the current layer thickness, and update array A. If array A is updated, jump to Step 2; otherwise, go to the next step.

Step 5: Arrange the triangular patches in array A updated by Step 4 to obtain the ring model of the triangular patches at the current layer, and then perform redundant removal work on the triangular patches; that is, delete the even-numbered triangular patches to update array A. As such, when determining the contour information, the calculation amount is reduced by half, and the running speed is improved.

Step 6: According to the updated array A in Step 5, calculate the current layer contour information point with the optimal thickness *h* determined in Step 3, and store it in the layered contour array.

Step 7: Judge whether the layering is over. If the layered slice is not completed, skip to Step 1 and continue to the next layer of slicing; otherwise, end the layering process.

**Figure 6:**
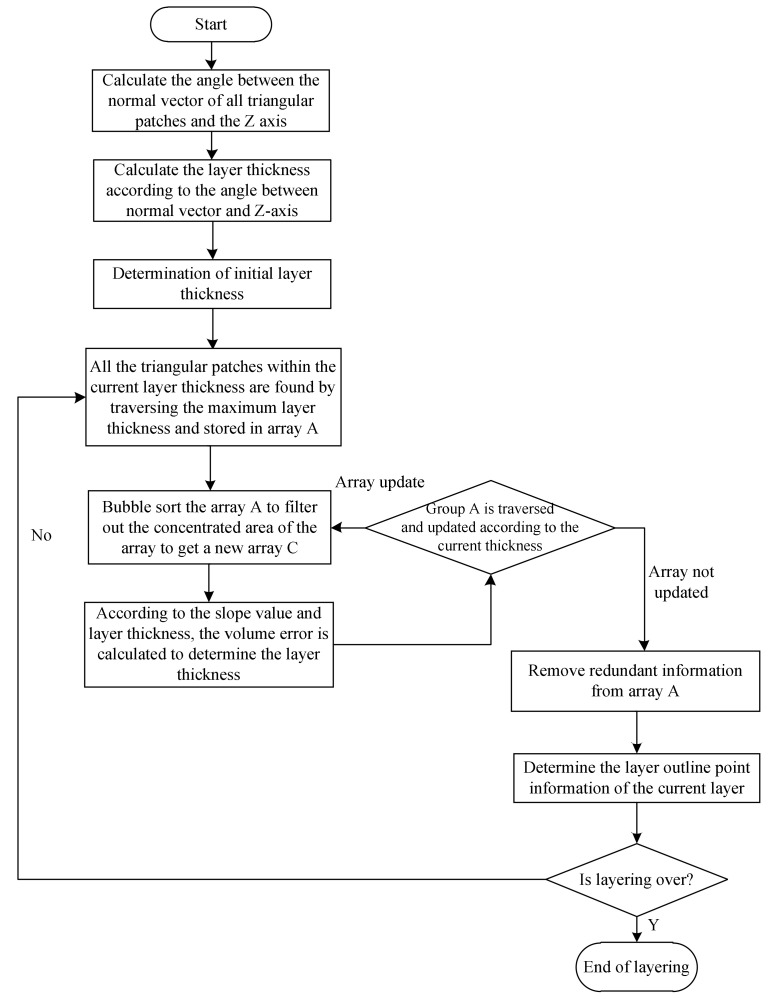
Adaptive hierarchical algorithm flow based on volume optimization.

The algorithm implementation process based on the optimal volume error is shown in Figure 6.

## 5. Experimental Research and Discussion

**Figure 7:**
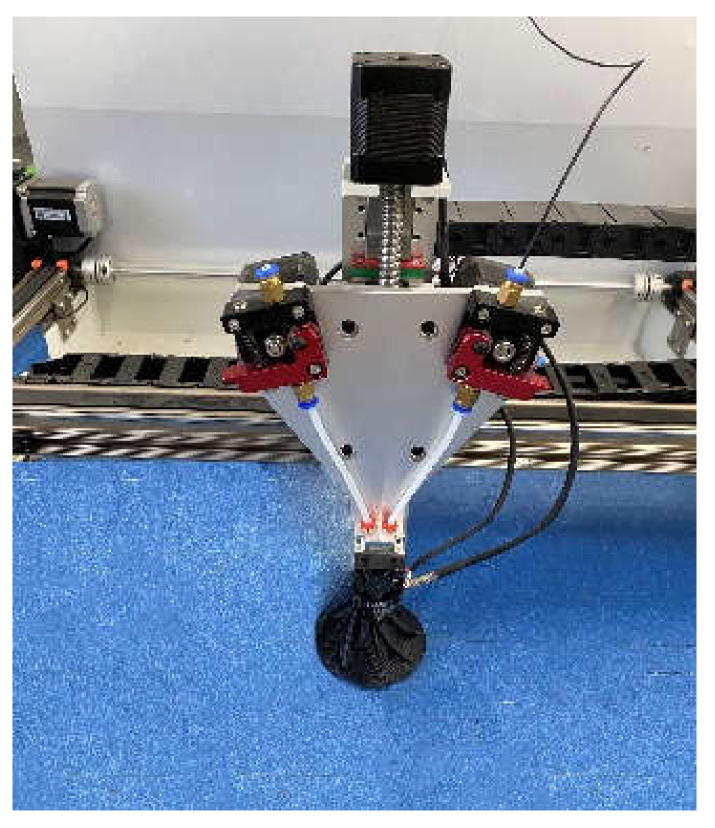
Experimental printing device.

**Figure 8:**
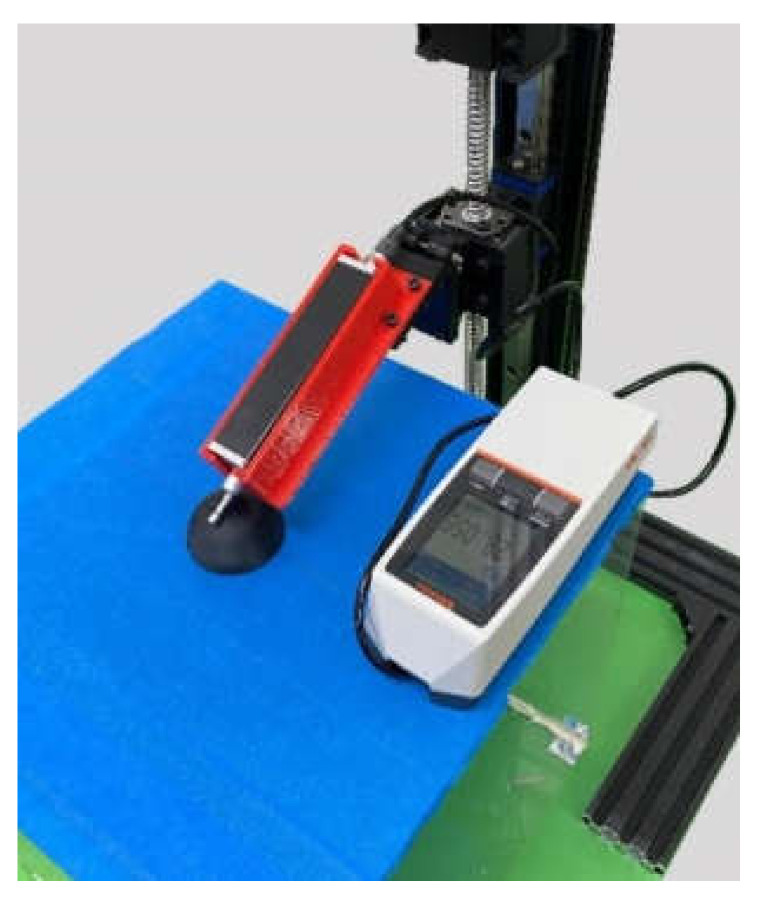
Measuring device diagram.

Based on Cura Engine open source code, Visual Studio2017 and C++ language are used to implement the algorithm in this paper, and then they are embedded into the FDM-3D printer control system to test the algorithm performance. An XYZ FDM-3D machine is used in the experiment, and the device is shown in Figure 7. The printing model material is PLA plastic, and the same printing parameters are used in different algorithms. The temperature of the extrusion head is 190 °C, the diameter of the nozzle is 0.6 mm, the wall thickness is 1.2 mm, the printing speed of the extrusion head is 50 mm/s, the no-load moving speed is 60 mm/s, the filling rate is 30%, the filling method is grid filling, and the filling line spacing is 3 mm. An Sj-210 surface roughness measuring instrument is used to measure the surface roughness of the printed test products. The testing device is shown in Figure 8.

**Figure 9:**
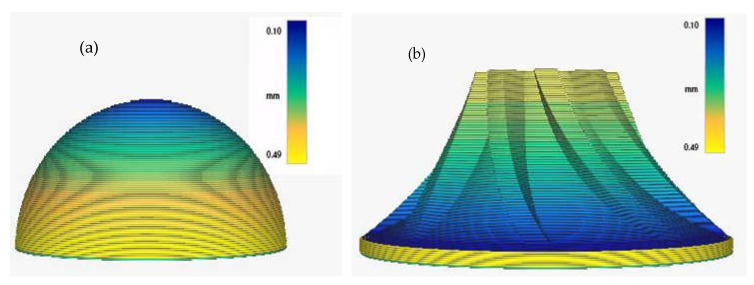
Optimal volumetric slices. (**a**) Hemispherical model. (**b**) Turbine blade model.

Two groups of models are selected for the test. Model 1 is a hemispherical model with triangular patches at any angle on the surface to test the adaptability of the algorithm. The radius of the hemispherical model is 15 mm. Model 2 is a turbine blade with a large radius of 25 mm, a small radius of 10 mm, and a side surface radius of 35 mm. The outer surface of the hemisphere is a convex spherical structure, while the turbine blade is a concave spherical structure, which more comprehensively verifies the influence of the layering algorithm on the surface accuracy of the model. Selecting the turbine blade model allows the complex model to be processed, which is closer to engineering practice and confirms the feasibility of the algorithm. The optimal volume error layering slices of the two test models are shown in Figure 9. It can be seen that the algorithm has good adaptability to the layering thickness in different regions of the model. The layer thickness is smaller in the area where the model accuracy is required to be high, while it is larger in the area where the model accuracy is low.

**Figure 10:**
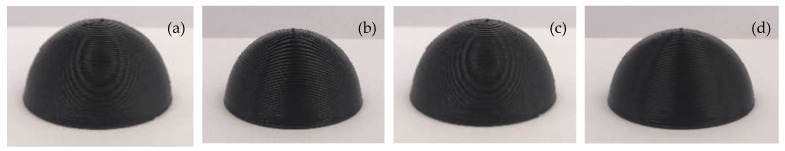
Finished parts of hemispherical model. (**a**) Finished part with slicing thickness of 0.4 mm. (**b**) Finished part with slicing thickness of 0.3 mm. (**c**) Finished part with slicing thickness of 0.2 mm. (**d**) Finished part manufactured by adaptive layering based on optimal volume error.

**Figure 11:**
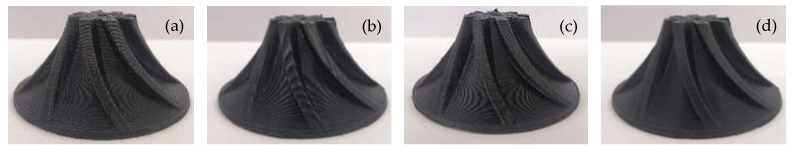
Finished parts of turbine blade model. (**a**) Finished part with slicing thickness of 0.4 mm. (**b**) Finished part with slicing thickness of 0.3 mm. (**c**) Finished part with slicing thickness of 0.2 mm. (**d**) Finished part manufactured using adaptive layering based on optimal volume error.

In the experiment, the algorithm presented in this paper is compared with the equal-thickness layered slices, which are 0.2 mm, 0.3 mm, and 0.4 mm. The slice thickness interval of the optimal volume error algorithm is set as [0.1, 0.5] mm. The G codes generated by the different algorithms are imported into the 3D printer control system to print the parts. The printed parts are shown in Figure 10; Figure 11, in which Figure 10a–c and Figure 11a–c display the equal thickness slicing Figure 10d and Figure 11d display the optimal volume error adaptive slicing. The layer thickness in Figure 10a and Figure 11a is 0.4 mm, the layer thickness in Figure 10b and Figure 11b is 0.3 mm, and the layer thickness in Figure 10c and Figure 11c is 0.2 mm; Figure 10d and Figure 11d display the finished parts printed by adaptive layering based on the optimal volume error.

**Figure 12:**
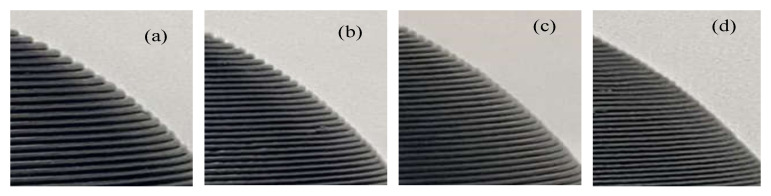
Partial details of the hemispherical model. (**a**) Slicing thickness is 0.4 mm. (**b**) Slicing thickness is 0.3 mm. (**c**) Slicing thickness is 0.2 mm. (**d**) Partial detail of adaptive layering based on optimal volume error.

**Figure 13:**
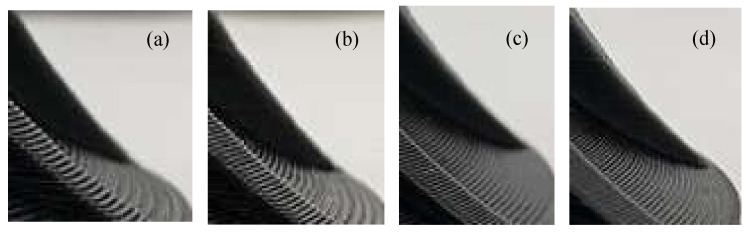
Partial details of turbine blade model. (**a**) Slicing thickness is 0.4 mm. (**b**) Slicing thickness is 0.3 mm. (**c**) Slicing thickness is 0.2 mm. (**d**) Partial detail of adaptive layering based on optimal volume error.

A partial enlargement of the printed hemispherical experimental part is shown in Figure 12, in which Figure 12a–c correspond to the partial enlarged views of the equal-thickness layering method in Figure 10a–c and Figure 12d corresponds to the partially enlarged views of the optimal volume error method in Figure 10d. A partial enlargement of the printed turbine blade experimental part is shown in Figure 13, in which Figure 13a–c correspond to the partial enlarged views of the equal-thickness layering method in Figure 11a–c and Figure 13d corresponds to the partially enlarged views of the optimal volume error method in Figure 11d.

It can be seen that, with the adaptive layering algorithm based on the optimal volume error, the step effect of the printed products significantly weakened, and the restoration accuracy of the contour details is good.

The experimental data are shown in Table 1. Theoretically, the smaller the thickness of the layer, the smaller the volume error and the higher the precision of the part, but this improvement in precision comes at the expense of increasing printing time. The following conclusions can be drawn from the experimental data:

1. For the hemispherical model, the printing accuracy of the parts based on the optimal volume error adaptive layering algorithm is much higher than that of the equal-thickness layering method with 0.3 mm and 0.4 mm layering thicknesses, and close to that with a 0.2 mm equal layering thickness, but the printing time is cut by more than half.
2. For the turbine blades, the printing time of the proposed layering algorithm is almost the same as that of the equal-thickness layering method with a 0.3 mm layering thickness, but the printing accuracy improved by 38.1%, and the surface forming accuracy improved by about 57.6% compared with the 0.4 mm layering thickness.

In conclusion, after comparing the volume error of the printed parts of the two models, it could be seen that the volume error of the layered algorithm proposed in this paper is minimal, and the molding time greatly reduced on the premise of ensuring printing accuracy.

## 6. Conclusions

The step effect in the FDM-3D molding process leads to a decrease in the surface accuracy of printing parts, and traditional layering algorithms cannot take into account both accuracy and printing time. On the basis of an analysis of the characteristics of the FDM process, a calculation formula of the volume error caused by the step effect is deduced, and an adaptive layering algorithm is proposed based on the principle of the optimal overall volume error of the model, which can better solve the problem regarding the fact that forming accuracy and printing time cannot be taken into account at the same time. The effectiveness of the proposed algorithm is verified by experiments.

From the perspective of the FDM process, there are many factors that affect molding accuracy. In addition to the stratification caused by the step phenomenon, there are also the approximation of the model discretization process; the cooling shrinkage deformation of the liquid-solid two-phase material in the printing process; and the matching relationship between the diameter of the extrusion head, the temperature of the extrusion head, the speed of the extrusion head, and the influence of the environment, etc. In the process of forming, variations in the material cooling temperature field and stress field cause the problem of multi-variable and multi-parameter coupling, which has the greatest influence on the precision of the forming parts. Therefore, future research will focus on the control of the variable parameters in the molding process.

## Figures and Tables

**Table 1:** Experimental measurement data.

Test Model	Layering Method	Layering Thickness (mm)	Molding Time (min)	Number of Layers	Volume Error (mm^3^)	Roughness *Ra* (μm)
Hemisphere	Equal-thickness layering method	0.2	97	124	128.83	17.3
		0.3	49	83	192.24	37.6
		0.4	34	62	251.81	52.4
	Our method	[0.1, 0.5]	44	81	105.24	21.3
Turbine blade	Equal-thickness layering method	0.2	169	174	255.12	25.3
		0.3	127	116	315.54	43.2
		0.4	107	87	419.15	63.1
	Our method	[0.1, 0.5]	128	122	224.62	26.7

## Data Availability

Anyone who needs the data, please ask the authors for it.

## References

[1] Dilberoglu U.M., Gharehpapagh B., Yaman U., Dolen M. (2017). The Role of Additive Manufacturing in the Era of Industry 4.0. Procedia Manuf..

[2] Abdulhameed O., Al-Ahmari A., Ameen W., Mian S.H. (2019). Additive manufacturing: Challenges, trends, and applications. Adv. Mech. Eng..

[3] Haleem A., Javaid M. (2019). Additive Manufacturing Applications in Industry 4.0: A Review. J. Ind. Integr. Manag..

[4] Kerekes T.W., Lim H., Joe W.Y., Yun G.J. (2018). Characterization of process–deformation/damage property relationship of fused deposition modeling (FDM) 3D-printed specimens. Addit. Manuf..

[5] Melocchi A., Uboldi M., Cerea M., Foppoli A., Maroni A., Moutaharrik S., Palugan L., Zema L., Gazzaniga A. (2020). A Graphical Review on the Escalation of Fused Deposition Modeling (FDM) 3D Printing in the Pharmaceutical Field. J. Pharm. Sci..

[6] García-Dominguez A., Claver J., Sebastián M.A. (2020). Integration of Additive Manufacturing, Parametric Design, and Optimization of Parts Obtained by Fused Deposition Modeling (FDM). A Methodological Approach. Polymers.

[7] Zhang X., Fan W., Liu T. (2020). Fused deposition modeling 3D printing of polyamide-based composites and its applications. Compos. Commun..

[8] Kristiawan R.B., Imaduddin F., Ariawan D., Arifin U., Arifin Z. (2021). A review on the fused deposition modeling (FDM) 3D printing: Filament processing, materials, and printing parameters. Open Eng..

[9] Nadiyapara H.H., Pande S. (2016). A Review of Variable Slicing in Fused Deposition Modeling. J. Inst. Eng. Ser. C.

[10] Wang M. (2018). Research on Direct Layering and Path Planning Technology of Additive Manufacturing. Mech. Eng. Autom..

[11] Jin Y.-A., Li H., He Y., Fu J.-Z. (2015). Quantitative analysis of surface profile in fused deposition modelling. Addit. Manuf..

[12] Sandhu G.S., Boparai K.S., Sandhu K.S. (2022). Effect of slicing parameters on surface roughness of fused deposition modeling prints. Mater. Today Proc..

[13] Sandhu G.S., Boparai K.S., Sandhu K.S. (2022). Influence of slicing parameters on selected mechanical properties of fused deposition modeling prints. Mater. Today Proc..

[14] Lieneke T., Denzer V., Adam G.A., Zimmer D. (2016). Dimensional Tolerances for Additive Manufacturing: Experimental Investigation for Fused Deposition Modeling. Procedia CIRP.

[15] Gong Y., Chen C., Xia M., Song E. (2016). Analysis of the step effect on the surface of the FDM 3D printed model. Manuf. Technol. Mach. Tool.

[16] Akhouri D., Banerjee D., Mishra S.B. (2020). A review report on the plating process of fused deposition modelling (FDM) built parts. Mater. Today Proc..

[17] Pu’ad N.M., Haq R.A., Noh H.M., Abdullah H., Idris M., Lee T. (2020). Review on the fabrication of fused deposition modelling (FDM) composite filament for biomedical applications. Mater. Today Proc..

[18] Nayyeri P., Zareinia K., Bougherara H. (2022). Planar and nonplanar slicing algorithms for fused deposition modeling technology: A critical review. Int. J. Adv. Manuf. Technol..

[19] Huang B., Singamneni S.B. (2015). Curved Layer Adaptive Slicing (CLAS) for fused deposition modelling. Rapid Prototyp. J..

[20] Chen S.M., Bai S.G. (2018). Adaptive Slicing Algorithm Based on Contour Line of CAD Model in 3D Printing. J. South China Univ. Technol..

[21] Li W.K., Chen C.B., Wu W.Y. (2015). Adaptive hierarchical algorithm for effectively retaining model features. Comput. Appl..

[22] Han J., Wang D.P., Xia L., Tian X.Q. (2020). Adaptive layering method to prevent feature shift of 3D printing model. J. Hefei Univ. Technol..

[23] Zheng H.L., Wang H.Y. (2017). Adaptive layering method for effectively retaining model features in rapid prototyping. Appl. Opt..

[24] Lv N., Zheng J., Zhao X., Xu W. (2019). Analysis and Optimization of HBP Temperature Field for FDM Rapid Forming Machine. China Mech. Eng..

